# Immunomodulation of dendritic cells by *Lactobacillus reuteri* surface components and metabolites

**DOI:** 10.14814/phy2.14719

**Published:** 2021-01-19

**Authors:** Melinda A. Engevik, Wenly Ruan, Magdalena Esparza, Robert Fultz, Zhongcheng Shi, Kristen A. Engevik, Amy C. Engevik, Faith D. Ihekweazu, Chonnikant Visuthranukul, Susan Venable, Deborah A. Schady, James Versalovic

**Affiliations:** ^1^ Department of Pathology and Immunology Baylor College of Medicine Houston TX USA; ^2^ Department of Pathology Texas Children’s Hospital Houston TX USA; ^3^ Department of Pediatrics Baylor College of Medicine Houston TX USA; ^4^ Section of Gastroenterology, Hepatology, and Nutrition Texas Children’s Hospital Houston TX USA; ^5^ Department of Neuroscience, Cell Biology and Anatomy University of Texas Medical Branch Galveston TX USA; ^6^ Department of Molecular Virology & Microbiology Baylor College of Medicine Houston TX USA; ^7^ Department of Surgery Vanderbilt University Medical Center Nashville TN USA; ^8^ Pediatric Nutrition Research Unit Department of Pediatrics Faculty of Medicine Chulalongkorn University Bangkok Thailand

**Keywords:** cytokines, dendritic cells, inflammation, *Lactobacillus*, metabolites

## Abstract

**Background:**

Lactic acid bacteria are commensal members of the gut microbiota and are postulated to promote host health. Secreted factors and cell surface components from *Lactobacillus* species have been shown to modulate the host immune system. However, the precise role of *L*. *reuteri* secreted factors and surface proteins in influencing dendritic cells (DCs) remains uncharacterized.

**Hypothesis:**

We hypothesize that *L*. *reuteri* secreted factors will promote DC maturation, skewing cells toward an anti‐inflammatory phenotype. In acute colitis, we speculate that *L*. *reuteri* promotes IL‐10 and dampens pro‐inflammatory cytokine production, thereby improving colitis.

**Methods & Results:**

Mouse bone marrow‐derived DCs were differentiated into immature dendritic cells (iDCs) via IL‐4 and GM‐CSF stimulation. iDCs exposed to *L*. *reuteri* secreted factors or UV‐irradiated bacteria exhibited greater expression of DC maturation markers CD83 and CD86 by flow cytometry. Additionally, *L*. *reuteri* stimulated DCs exhibited phenotypic maturation as denoted by cytokine production, including anti‐inflammatory IL‐10. Using mouse colonic organoids, we found that the microinjection of *L*. *reuteri* secreted metabolites and UV‐irradiated bacteria was able to promote IL‐10 production by DCs, indicating potential epithelial‐immune cross‐talk. In a TNBS‐model of acute colitis, *L*. *reuteri* administration significantly improved histological scoring, colonic cytokine mRNA, serum cytokines, and bolstered IL‐10 production.

**Conclusions:**

Overall these data demonstrate that both *L*. *reuteri* secreted factors and its bacterial components are able to promote DC maturation. This work points to the specific role of *L*. *reuteri* in modulating intestinal DCs.

**New & Noteworthy:**

*Lactobacillus* *reuteri* colonizes the mammalian gastrointestinal tract and exerts beneficial effects on host health. However, the mechanisms behind these effects have not been fully explored. In this article, we identified that *L*. *reuteri* ATTC PTA 6475 metabolites and surface components promote dendritic cell maturation and IL‐10 production. In acute colitis, we also demonstrate that *L*. *reuteri* can promote IL‐10 and suppress inflammation. These findings may represent a crucial mechanism for maintaining intestinal immune homeostasis.

## Graphical abstract

*L*. *reuteri* secreted factors and its bacterial components are able to promote DC maturation and IL‐10 production. Additionally, *L*. *reuteri* is able to elevate IL‐10 and suppress inflammation in a TNBS model of colitis.

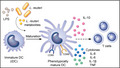

**Abbreviations**
CM: conditioned media
DC: dendritic cells
IF: immunofluorescence
LDM4: fully defined media

## 1 INTRODUCTION

Dendritic cells (DCs) are migratory phagocytic cells that act as the gatekeepers of the immune system (Cella et al., 1997; Hammer & Ma, 2013; Kelsall et al., 2002; Kelsall & Strober, 1997; Mowat et al., 2003). Intestinal DCs mediate tolerance to commensal microbes and food antigens, while propagating the appropriate response to harmful pathogens. The distinct properties of DCs are influenced by both host and microbial signals. Intestinal DCs survey the microenvironment via antigen uptake and respond to environmental cues. The ability of DCs to regulate intestinal immunity largely depends on their maturation (Banchereau et al., 2000; Cella et al., 1997; Cresswell, 1994; Garrett et al., 2000; Hammer & Ma, 2013; Kamath et al., 2000; Kelsall et al., 2002; Kelsall & Strober, 1997; Mohamadzadeh et al., 2005; Reis e Sousa, 2006; Trombetta et al., 2003; Turley et al., 2000). Immature DCs are recruited to sites of inflammation and then migrate to T cell‐rich areas within lymphoid organs after acquiring the appropriate stimuli. In the lymphoid organs, DCs undergo maturation and modulate their cytokine expression profiles. Programed maturation enhances the ability of DCs to activate other immune cells (Banchereau et al., 2000). Mature DCs promote the polarization of naïve T cells toward Th1, Th2, Th17, or T regulatory (Treg) cell responses (Banchereau et al., 2000; Rescigno & Di Sabatino, 2009). Of particular interest, primed Tregs and DCs produce the anti‐inflammatory cytokine IL‐10 and suppress inflammation (Powrie et al., 2003; Rescigno & Di Sabatino, 2009). Thus, DCs can orchestrate intestinal responses and maintain homeostasis.

Commensal intestinal bacteria modulate the immune system. Work in germ‐free mice demonstrates that the gut microbiota is required for immune maturation and proper inflammatory responses (Atarashi et al., 2011; Duan et al., 2010; Mazmanian et al., 2008; Round & Mazmanian, 2009). DCs harbor a diverse array of microbial sensors, including pattern recognition receptors (PRRs) of the Toll‐like receptors (TLR), which are critical for DC maturation (Meijerink et al., 2012). In addition to direct the activation of TLRs through cell surface components, commensal microbes also secrete a variety of immunomodulators, including outer membrane vesicles (OMVs), short‐chain fatty acids (SCFAs), long‐chain fatty acids (LCFAs), and polysaccharides that can also influence DCs (Engevik & Versalovic, 2017; Owen & Mohamadzadeh, 2013; Saemann et al., 2002; Uribe‐Herranz et al., 2020; Whiteson et al., 2017). *Lactobacilli* are a major component of the mammalian commensal microbiota and are considered to be probiotic because they provide health benefits without causing disease (Guarner & Schaafsma, 1998; Hill et al., 2014; Klaenhammer & Kullen, 1999). Species of *Lactobacilli* have been shown to modulate DC properties (Ahrne et al., 1998; Braat, van den Brande, et al., 2004; Braat, de Jong, et al., 2004; Cai et al., 2016; Christensen et al., 2002; Elawadli et al., 2014; Evrard et al., 2011; Gorska et al., 2014; Haileselassie et al., 2016; Hart et al., 2004; Jo et al., 2016; Karlsson et al., 2004; Mohamadzadeh et al., 2005; Tang et al., 2015; Yang et al., 2015; You et al., 2014; Zeuthen et al., 2006), but the mechanisms through which these species alert the immune system remains unclear. Moreover, probiotic effects appear to be strain‐specific, highlighting the need to define the precise effects of individual bacterial strains. We selected *L*. *reuteri*, a human gut commensal (Walter et al., 2011), which is tolerated, safe, and efficacious in infants, children, and adults (Hoy‐Schulz et al., 2016; Jones et al., 2012; Jones & Versalovic, 2009; Mangalat et al., 2012; Mu et al., 2018; Valeur et al., 2004; Weizman & Alsheikh, 2006). Multiple studies have demonstrated that *L*. *reuteri* can induce anti‐inflammatory Treg cells and suppress Th1/Th2 responses (He et al., 2017; Karimi et al., 2009; Liu et al., 2013; Livingston et al., 2010; Poutahidis, Kearney, et al., 2013; Poutahidis, Kleinewietfeld, et al., 2013; Sims et al., 2011); effects which are speculated to contribute to the beneficial influence of *L*. *reuteri* on the host. However, the effects of *L*. *reuteri* on DCs are underexplored. Since DCs prime T‐cells, these initial interactions likely mediate anti‐inflammatory responses. As active bacterial metabolites can cross intestinal epithelial monolayers (Hoarau et al., 2006; Menard et al., 2004), we hypothesized that *L*. *reuteri* secreted products and cell wall components will mature DCs, skewing their interactions toward anti‐inflammatory immune responses. We, therefore, studied the effects of secreted metabolites from *L*. *reuteri*, as well as heat‐killed bacteria, on DCs to define the interactions of this probiotic strain with the immune system and investigated its anti‐inflammatory effect in mice with 2,4,6‐trinitrobenzene sulfonic acid (TNBS)‐induced colitis.

## 2 METHODS

### 2.1 Bacterial growth conditions

*Lactobacillus* *reuteri* ATCC‐PTA 6475 was previously isolated from feces (Mu et al., 2018). *L*. *reuteri* was stored at −80°C in de Man, Rogosa, and Sharpe media (MRS) (Difco, Franklin Lakes, NJ) supplemented with 20% glycerol, and sub‐cultured in MRS agar and broth under anaerobic conditions in an anaerobic workstation (Anaerobe Systems AS‐580) supplied with a mixture of 5% CO_2_, 5% H_2_, and 90% N_2_ at 37 °C before use. Following MRS growth, *L*. *reuteri* was sub‐cultured in a fully defined media, termed LDM4 (Engevik, Morra, et al., 2019) for all experiments. Briefly, *L*. *reuteri* at the exponential phase in MRS was used to inoculate LDM4, at OD_600 nm_ adjusted to 0.1 and incubated anaerobically at 37°C overnight. To generate *L*. *reuteri* conditioned media, stationary phase (~16 h) bacterial cells were centrifuged at 5000 × *g* for 10 min to remove bacteria and the supernatant pH was adjusted to pH 7.0. Then the supernatant was filtered through a 0.2 µm membrane filter (Millipore # SLGV033RS). As a negative control, the un‐inoculated LDM4 bacterial medium underwent the same process.

### 2.2 Generation of mouse bone marrow‐derived dendritic cells

All experimental protocols were approved by the Baylor College of Medicine Animal Care and Use Committee and complied with National Institutes of Health guidelines. BALB/c male mice (8–12 weeks old) were purchased from Taconic, housed at Baylor College of Medicine. Male mice were used for bone marrow isolation since cytokine production has been shown to differ between male and female DCs (Butts et al., 2008). To isolate bone marrow cells, male BALB/c mice were euthanized according to an approved protocol and processed as previously described (Matheu et al., 2008). Briefly, the femur and tibia were flushed with RPMI‐1640 cell culture media, centrifuged, and treated with red blood cell lysis buffer. Following additional washing, 10^5^ mL^−1^ bone marrow cells were seeded into 10 cm Petri dishes in RPMI‐1640 supplemented with 10% (v/v) heat‐inactivated FBS, 100 ng/mL murine IL‐4 (PeproTech #214‐14), and 100 ng/mL murine GM‐CSF (PeproTech #315‐03). Mouse bone marrow cells were incubated at 37°C, 5% CO_2_ for 7 days with a media change on day 3. For experiments, DCs were trypsinized and seeded in new quadrant Petri dishes at 2 × 10^5^ cells/mL. On average, DC cell viability was determined to be 97% ±4.5 by trypan blue staining using the Invitrogen Countess (Invitrogen). DCs were treated with either 25% un‐inoculated LDM4 (media), 100 ng/mL of lipopolysaccharide (LPS) (derived from *Escherichia* *coli* O111:B4; InvivoGen #tlrl‐3pelps), 25% *L*. *reuteri* LDM4 conditioned medium or 10^6^ CFU (colony forming units) of UV‐irradiated *L*. *reuteri* bacteria overnight. After the incubation, the supernatant was collected for cytokine analysis and the cells were collected in TRIZOL for RNA analysis. Alternatively, DCs were harvested, washed, and used for cytometric analysis or functional assays.

### 2.3 Resazurin assay

The resazurin assay was used to assess the metabolic activity of treated DCs, as previously described (Feng & Cohen, 2013). Briefly, resazurin (7‐hydroxy‐3H‐phenoxazin‐3‐one 10‐oxide) (Sigma # R7017) was added to each treatment group at a final concentration of 44 µM and incubated for 4 h at 37°C. The fluorescent conversion of resazurin to resorufin was examined with a microplate spectrofluorometer at an excitation wavelength of 570 nm and an emission wavelength of 600 nm. Metabolic activity was calculated by comparing readings for treatment groups against those obtained for wells incubated only with RPMI +inoculated LDM4 medium.

### 2.4 Flow cytometry

For flow cytometry analysis, 2 x10^5^ DCs were harvested by centrifugation, washed once in 2% FBS in PBS (Life Technologies) then resuspended in 100 µL 2% FBS in PBS. One microliter of FC blocker (BD Biosciences # 555404) was added to each sample and incubated for 15 min on ice. Sample viability was assessed using Fixable Viability Dye eFluor™ 780 (Thermo Fisher # 65–0865–14). Cells were then incubated with saturating concentrations of the different fluorochrome‐conjugated monoclonal antibodies (FITC‐conjugated anti‐CD86, APC‐conjugated CD80, PE‐conjugated anti‐CD11c, and PE‐Cy7‐conjugated anti‐MHCII (BD Biosciences) for 30 minutes at 4°C. The stained cells were washed twice in PBS and fixed in 1% paraformaldehyde PBS solution until analysis by flow cytometry. Flow cytometry was performed with the BD FACS Canto flow cytometer. Data were collected with FACS Diva software and analyzed using FlowJo V10. Cells were analyzed for geometric mean fluorescence intensity (mean fluorescence intensity of the antibody of interest/mean fluorescence intensity of isotype control) and for the percentage of marker‐positive cells. Over 10,000 cells per sample were analyzed.

### 2.5 Mouse organoid model

Mouse organoids were generated as previously described (Fernando et al., 2017). Briefly, the ileum was removed from 8 to 12‐week‐old male BALB/c mice and washed thoroughly in ice‐cold Ca^2+^/Mg^2+^‐free DPBS. After washing and cutting into small sections, the tissue was incubated in 3 mM EDTA, DTT, and sucrose at 4 ºC for 30 min. Crypts were collected in chelation buffer (sucrose, sorbitol, and bovine serum albumin), centrifuged at 300 × g for 10 minutes, and embedded in Matrigel (BD Biosciences). After Matrigel polymerization, Matrigel domes were covered with CMGF+media (purchased from the BCM GEMS Core) without Wnt containing 10 µM Y‐27632 rock inhibitor (Chang‐Graham et al., 2019). Organoids were used at passage 3 to ensure the remaining debris was removed. After 5 days of growth, organoids were microinjected with 17.6 nl of solution (uninoculated LDM4 media control, 100 ng/mL of LPS or *L*. *reuteri* LDM4 conditioned media or *L*. *reuteri* UV‐irradiated bacteria) using a Nanoject microinjector (Drummond Scientific Company) as previously described (Engevik et al., 2013). Organoids were incubated overnight and the supernatant was analyzed using Luminex Multiplex Assay.

### 2.6 *L. reuteri* adhesion assays

Human mucin‐producing HT29‐MTX cells (Sigma # 12040401) were grown in Gibco Dulbecco's Modified Eagle Medium (Thermo Fisher) supplemented with 10% fetal bovine serum (FBS) at 37°C, 5% CO_2_ with frequent *Mycoplasma* testing (Lonza t# LT07‐518). For adhesion experiments, cells were seeded at 2 × 10^5^ cells/cm^2^ in 24‐well tissue culture‐treated plates (Corning) with Poly‐L‐lysine coated glass coverslips at the bottoms of the wells. Cells were grown until they reached confluence and were then incubated with CFDA‐SE tagged *L*. *reuteri* for 1 hr, as previously described (Engevik, Luk, et al., 2019). Cells were then thoroughly rinsed and fixed. Cells for Scanning Electron Microscopy were fixed in 2.5% glutaraldehyde in PBS for 1 hr at room temperature, dehydrated, coated in 20 nm of gold (Denton Desk II), and viewed using a scanning electron microscope (FEI XL‐30FEG) at 12 kV as previously described (Engevik, Luk, et al., 2019). Cells for immunostaining were fixed in Clark's fixative (to preserve the mucus architecture) for 1 hr at room temperature. Immunostaining was performed by permeating the cells with 0.1% Triton‐X (Sigma) in PBS for 30 min at room temperature, blocking with 10% normal donkey serum (Sigma), and incubation with an anti‐MUC2 antibody (dilution: 1:200, Santa Cruz #sc‐15334) overnight at 4°C. Cells were then thoroughly washed and incubated with donkey‐anti‐rabbit‐Alexa Fluor 564 diluted at 1:1,000 (Life Technologies) for 1 hr at room temperature. To stain the nuclei, Hoechst 33342 (Invitrogen) was incubated at room temperature for 10 min. Coverslips were mounted on glass slides with Prolong antifade mounting media (Life Technologies). Slides were imaged on an upright widefield epifluorescence Nikon Eclipse 90i (Nikon) with a 20x Plan Apo (NA 0.75) differential interference contrast (DIC) objective and a CoolSNAP HQ2 camera (Photometrics) with a SPECTRA × LED light source (Lumencor).

### 2.7 TNBS colitis model

For TNBS animal experiments, BALB/c mice (8–12 weeks old), both male and female, were purchased from Taconic, housed at Baylor College of Medicine, and pre‐treated by oral gavage with 10^9^ CFU mL^−1^ *L*. *reuteri* in PBS or sterile PBS (Thermo Fisher) daily for 1 week. To administer 2,4,6‐Trinitrobenzenesulfonic acid (TNBS), mice were anesthetized by isoflurane inhalation and 5% (wt/vol) TNBS in ethanol was rectally delivered. Mice received daily oral gavage of treatment (*L*. *reuteri* in PBS or sterile PBS) until euthanasia (3 days). Cardiac punctures were used to obtained serum. The cecum and colon were removed, fixed in Carnoy's fixative, and then processed for H&E and Giemsa staining. Histological scores of colitis were assessed from H&E stained slides by a board‐certified anatomic pathologist.

Mouse colon was isolated and homogenized in TRIZOL to obtain RNA. RNA was isolated according to the manufacturer's details (Thermo Fisher # 15596018) and 1 μg RNA was converted to cDNA using the SensiFAST cDNA synthesis kit (Bioline USA Inc). Quantitative real‐time PCR (qPCR) was performed using Fast SYBR Green (Thermo Fisher) with primers designed using Primerdesign (Thermo Fisher) on a QuantStudio 3 qPCR machine (Applied Biosystems). Data are presented as the relative fold change calculated using the ΔddCT method with the housekeeping gene 18S.

### 2.8 MAGPIX protein analysis

Mouse serum and DC supernatant samples were examined by MAGPIX protein analysis according to the manufacturer's details. Samples were assayed using a MILLIPLEX Magnetic Bead Panel, cat. # MCYTOMAG‐70 K (Millipore) with a MAGPIX instrument (Luminex Corporation) by the Texas Medical Center Digestive Disease Center Core. The analytes tested included IL‐8, TNF, IL‐1β, IL‐12 (p70), and IL‐6. Luminex xPONENT for MAGPIX, version 4.2 Build 1324, and MILLIPLEX Analyst version 5.1.0.0 standard Build 10/27/2012 were used to analyze the raw data. IL‐10 was measured by ELISA (Thermo Fisher #88‐7105–22).

### 2.9 Statistics

Examinations of data between groups were made with either student t‐test, One‐way or Two‐way Analysis of Variance (ANOVA), and the Holm‐Sidak post hoc test using SigmaPlot (Systat Software, Inc., San Jose, CA). Graphs were created using GraphPad (GraphPad Software, Inc. La Jolla, CA). A *p* < 0.05 value was considered significant, while *n* is the number of experiments performed.

## 3 RESULTS

### 3.1 *Lactobacillus reuteri* modulates mouse bone‐marrow‐derived DC phenotypes

**FIGURE 1:**
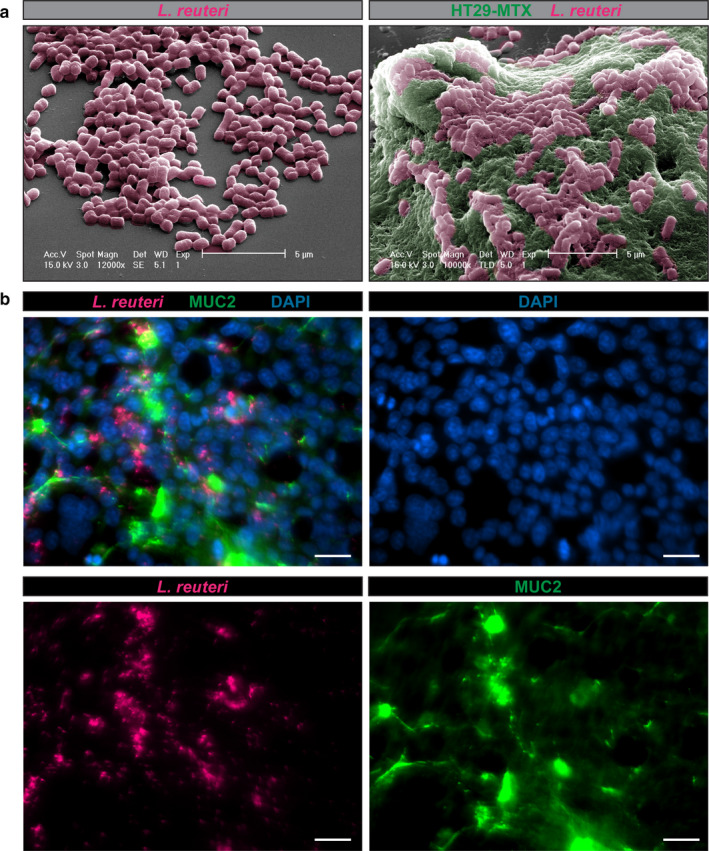
*L*. *reuteri* ATCC PTA 6475 adheres to human MUC2. a. Scanning electron microscopy (SEM) image of *L*. *reuteri* on a membrane (left) and *L*. *reuteri* adhered to mucus in human mucus‐producing HT29‐MTX cells. Scale bar =5 μm. b. Representative images of CFDA‐SE tagged *L*. *reuteri* (pink) co‐localizing with human HT29‐MTX MUC2 (green) after 1 hr incubation. Nuclei are marked with Hoechst dye (blue). Scale bar =100 μm. *n* = 3 replicates

Mucus‐associated gut commensals can adhere to and colonize the mucus of the gastrointestinal tract. Adherence to the intestinal mucus layer by probiotic bacteria is a particularly desirable feature as it likely increases gut residence time (Jensen et al., 2014; Juge, 2012; Kleerebezem et al., 2010; Lebeer et al., 2008). *Lactobacilli* contain several mucus binding proteins, including mucus‐binding protein (Mub), adhesion‐promoting protein MapA, surface protein CnBP, high‐molecular‐mass surface protein (Lsp), methionine sulfoxide reductase B (MsrB), and cell and mucus binding protein A (CmbA) (Aleljung et al., 1994; Jensen et al., 2014; Miyoshi et al., 2006; Rojas et al., 2002; Roos et al., 1996; Roos & Jonsson, 2002; Walter et al., 2011). In the intestinal mucus layer, microbes are in closer proximity to the host epithelium, where they can secrete factors capable of suppressing epithelial or immune cell pro‐inflammatory cytokines. As a result, mucus adhering microbes represent prime candidates for the targeted delivery of therapeutics metabolites. Using the mucus‐producing goblet cell line HT‐29‐MTX, we demonstrate that *L*. *reuteri* ATCC PTA 6475 adheres to human MUC2 mucus after 1 hr incubation as determined by immunofluorescence and scanning electron microscopy (Figure 1a, b).

**FIGURE 2:**
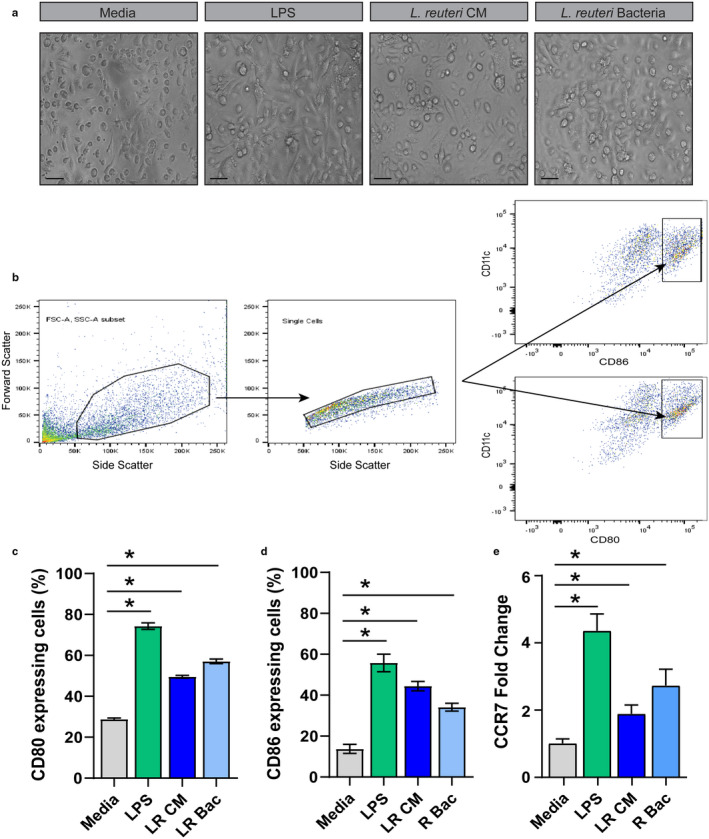
Modulation of mouse bone‐marrow dendritic cell surface markers by *L*. *reuteri*. a. Representative light microscopy images of DCs exposed to 25% uninoculated LMD4 (Media), 100 ng/mL of LPS (LPS), 25% *L*. *reuteri* LDM4 conditioned media (*LR* CM) or 10^6^ UV‐irradiated *L*. *reuteri* (*LR* bacteria). Scale bar =100 μm. b. Representative images of flow cytometry gating for mouse bone marrow‐derived dendritic cells. Flow cytometry was performed by gating CD11c^hi^ DC populations and examining CD80 (C) and CD86 (D) abundance on DCs exposed to 25% uninoculated LMD4 (Media), 100 ng/mL of LPS (LPS), 25% *L*. *reuteri* LDM4 conditioned media (*L*. *reuteri* CM) or 10^6^ UV‐irradiated *L*. *reuteri* (*L*. *reuteri* Bacteria). Surface marker abundance was expressed by % abundance for (c) CD80, (d) CD86 positive populations. e. qPCR analysis of CCR7 mRNA expression in treated DCs. *n* = 3 technical replicates, 4 mice/group. ANOVA, *p < 0.05

DCs may be targeted for modulation by gut microbes, including probiotic *Lactobacillus* species. *L*. *reuteri* secretes a variety of factors that are capable of modulating the host (Britton et al., 2014; Forsythe et al., 2007; Haileselassie et al., 2016; Jones & Versalovic, 2009; Thomas et al., 2012), but their effects on DCs are poorly characterized. To determine if *L*. *reuteri* secreted metabolites or cell surface proteins could induce DC maturation, we measured the abundance of maturation‐specific cell surface markers by flow cytometry. Mature DCs express high levels of CD80 and CD86 (Chang‐Graham et al., 2019), which are required to activate T cells. Immature mouse bone marrow‐derived DCs were incubated with 25% uninoculated LDM4 bacterial media (media), 25% *L*. *reuteri* LDM4 conditioned media (*L*. *reuteri* CM) or 10^6^ UV‐irradiated *L*. *reuteri* bacteria at 37°C overnight. As a positive control, DCs were also incubated with 100 ng/mL of LPS. As seen in Figure 2a, DCs were visualized by light microscopy and all treated DCs appeared normal with extensive dendrite formation. DCs were examined by flow cytometry with the gating of the integrin CD11c (Figure 2b). As expected, DCs treated with *E*. *coli* LPS upregulated the surface expression of the T‐cell costimulatory molecule CD80 and CD86 (Figure 2b‐d). *L*. *reuteri* conditioned media and heat‐killed *L*. *reuteri* bacteria also induced the upregulation of surface expression of the T‐cell costimulatory molecule CD80 and CD86 (Figure 2c‐d). Upon maturation, DCs upregulate C‐C Motif Chemokine Receptor 7 (CCR7) (Ricart et al., 2011). To determine if *L*. *reuteri* metabolites or cell components could influence CCR7 expression, we examined mRNA expression by qPCR (Figure 2e). *L*. *reuteri* conditioned media treated DCs exhibited a 1.9‐fold increase in CCR7, while UV‐irradiated *L*. *reuteri* treated DCs had a 2.7‐fold increase compared to media controls. Likewise, LPS treated DCs exhibited a 4.4‐fold increase in CCR7. These data indicate that *L*. *reuteri* metabolites and surface components can influence aspects of DC maturation.

**FIGURE 3:**
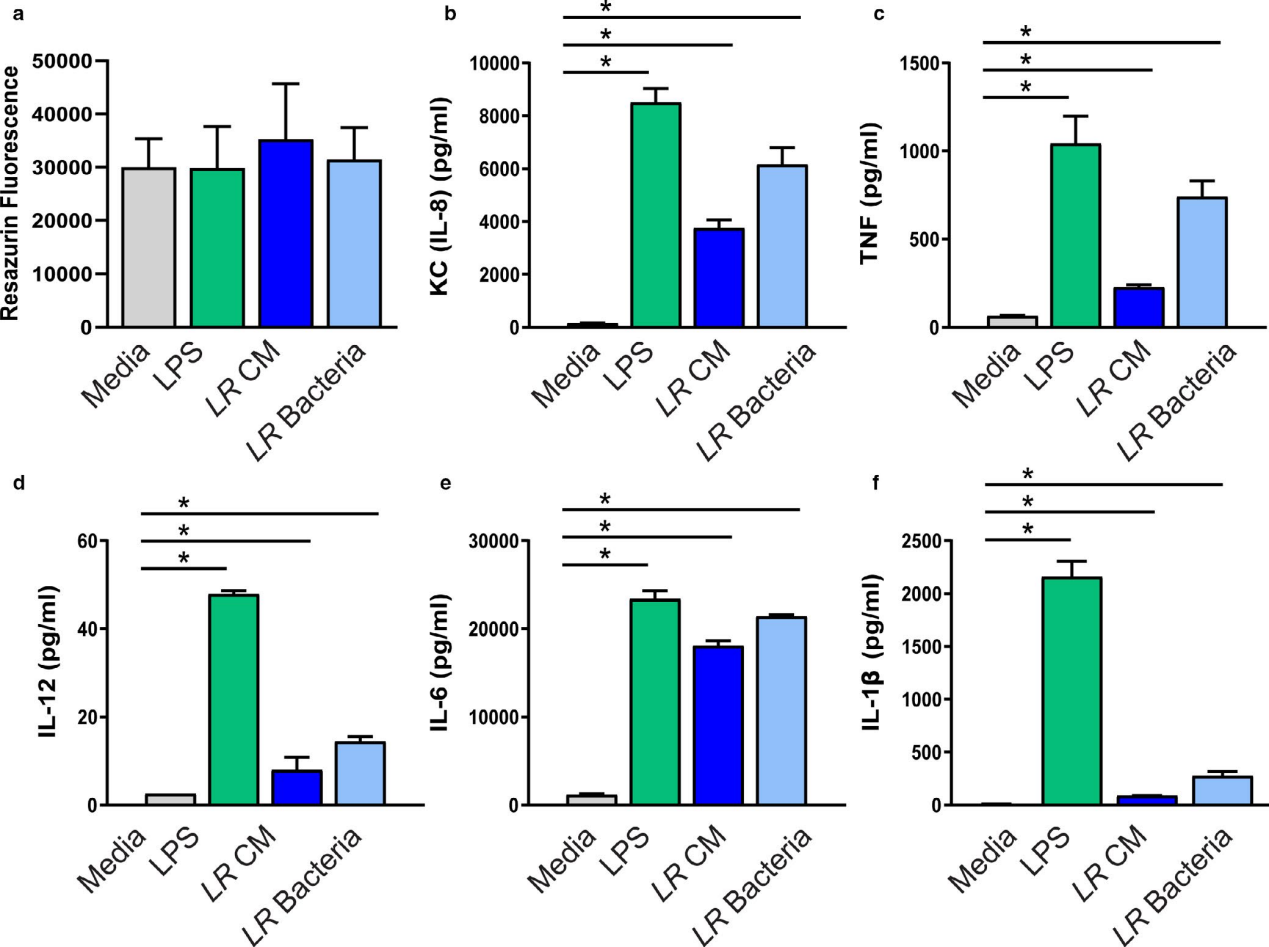
Modulation of mouse bone marrow‐derived dendritic cell cytokines by *L*. *reuteri*. a. DCs exposed to 25% uninoculated LMD4 (Media), 100 ng/mL of LPS (LPS), 25% *L*. *reuteri* LDM4 conditioned media (*LR* CM) or 10^6^ UV‐irradiated *L*. *reuteri* (*LR* bacteria). Scale bar =100 μm. Viability analysis of resazurin assay, measured by resorufin fluorescence (Ex:560 nm/Em:600 nm). Secreted cytokines from treated DCs after 16 hrs of treatment measuring: (b) KC (mouse IL‐8 homolog), (c) TNF, (d) IL‐12, (e) IL‐6, and (f) IL‐1β. *n* = 3 technical replicates, 4 mice/group. ANOVA, *p < 0.05

To confirm that our bacterial metabolites were not affecting viability, we examined DCs using the resazurin assay. Resazurin acts as an intermediate electron acceptor in the electron transport chain and can be reduced by NADPH and cytochromes. As resazurin accepts electrons, it changes from the oxidized, non‐fluorescent state to the reduced, fluorescent state. All treatment groups resulted in comparable resazurin fluorescence, indicating no changes to viability and metabolic function (Figure 3A). DCs can secrete several cytokines and mature DCs secrete IL‐12, IL‐6, IL‐1β, TNF, and KC (the mouse homolog to human IL‐8). To determine if our *L*. *reuteri* treatment promoted functional maturation, we examined these cytokines in the supernatants of DCs. Interestingly, we observed increased concentrations of all cytokines in response to both *L*. *reuteri* conditioned media as well as *L*. *reuteri* UV‐irradiated bacteria (Figure 3B‐F). As expected, our positive control *E*. *coli* LPS also promoted functional maturation, as seen by cytokine secretion. These findings demonstrate that *L*. *reuteri* metabolites and surface proteins are capable of eliciting phenotypic and functional changes in DCs.

### 3.2 *L. reuteri* promotes IL‐10 production

**FIGURE 4:**
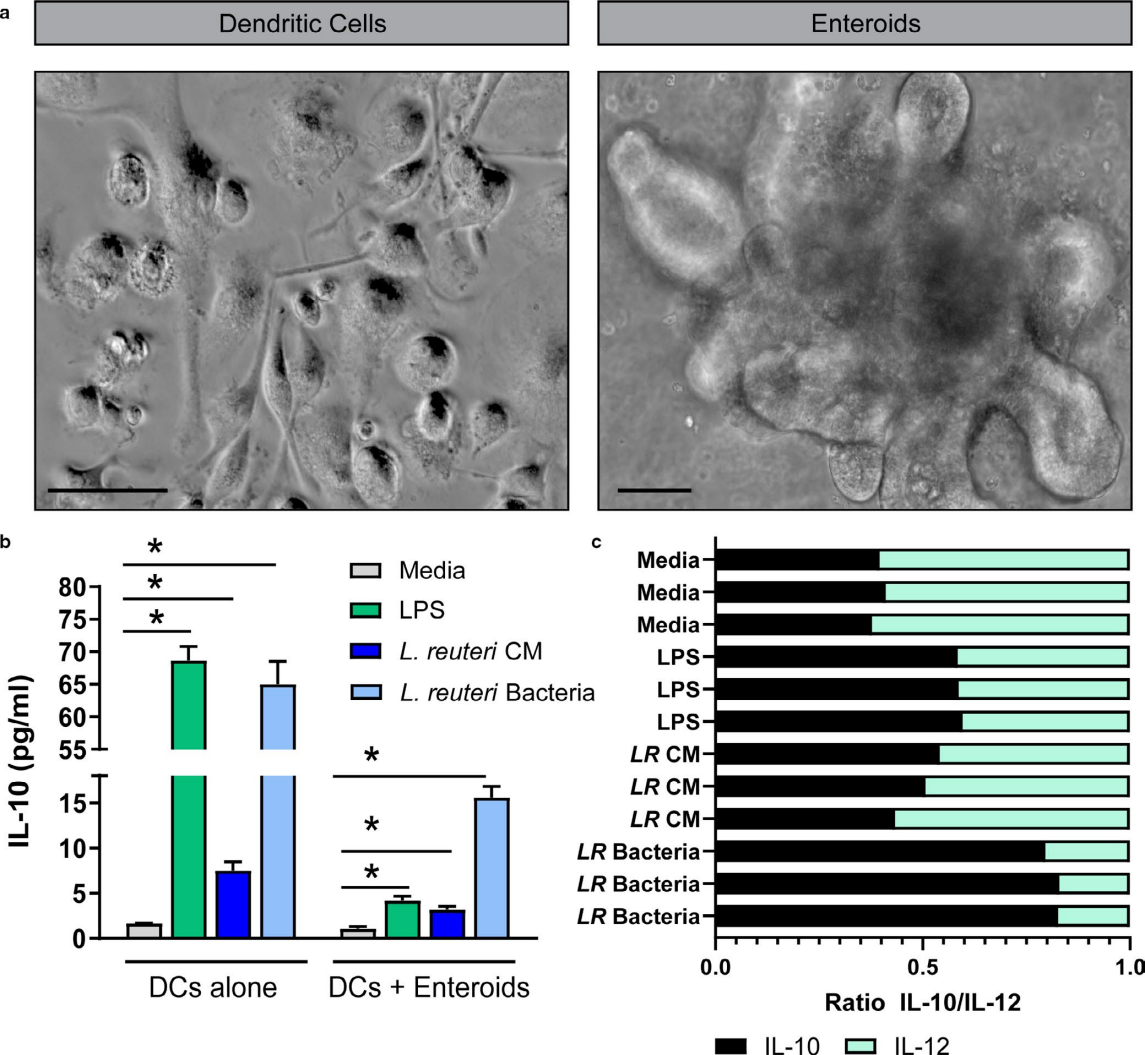
*L*. *reuteri* promotes IL‐10 production in vitro. a. Representative light microscopy images of mouse DCs and ileal organoids. Scale bar =100 μm. b. IL‐10 levels from treated DCs or DC/organoid co‐cultures after 16 hrs of treatment. c. IL‐10 to IL‐12 ratio for DCs treated with 25% uninoculated LMD4 (Media), 100 ng/mL of LPS (LPS), 25% *L*. *reuteri* LDM4 conditioned media (*LR* CM) or 10^6^ UV‐irradiated *L*. *reuteri* (*LR* bacteria). *n* = 3 technical replicates, 4 mice/group. ANOVA, *p < 0.05

Among the cytokines produced by DCs, interleukin 10 (IL‐10) is a key anti‐inflammatory cytokine that aids in preventing chronic inflammation and tissue damage (Schulke, 2018). We sought to assess whether *L*. *reuteri* metabolites and surface proteins could stimulate IL‐10 directly in DCs or indirectly through the intestinal epithelium. To achieve this, we measured IL‐10 by ELISA from the supernatant of DCs treated with *L*. *reuteri* conditioned media, UV‐irradiated *L*. *reuteri* bacteria, or LPS. Additionally, we microinjected ileal organoids derived from BALB/c mice with *L*. *reuteri* conditioned media, UV‐irradiated *L*. *reuteri* bacteria or LPS and co‐cultured the organoids with DCs. Examples of DCs and organoids can be seen in the light micrographs in Figure 4A. We found that the direct application of *L*. *reuteri* conditioned media, bacteria, and LPS stimulated the substantial secretion of IL‐10, with the highest levels observed with LPS and *L*. *reuteri* bacteria (Figure 4B). When organoids were microinjected with treatment, media levels of IL‐10 were significantly decreased, suggesting that an intact epithelial layer dampens the ability of metabolites and bacterial products to stimulate immune cells. However, we still observed increased IL‐10 in LPS, *L*. *reuteri* conditioned media, and bacteria treated organoids/DCs compared to media control, indicating that some cross‐talk can still occur. The ratio of IL‐10 to IL‐12 is often used as an indicator of the potential for DCs to polarize T cell responses toward either Th1 or Th2/Treg (Meijerink et al., 2012). Strikingly, we observed high IL‐10/IL‐12 ratios in DCs treated with *L*. *reuteri* bacteria compared to all other treatments (Figure 4C). These findings suggest that *L*. *reuteri* can beneficially modulate IL‐10 and that *L*. *reuteri* cell surface components may be more potent for IL‐10 stimulation than *L*. *reuteri* metabolites.

### 3.3 *L. reuteri* suppresses colitis and pro‐inflammatory cytokines in vivo

**FIGURE 5:**
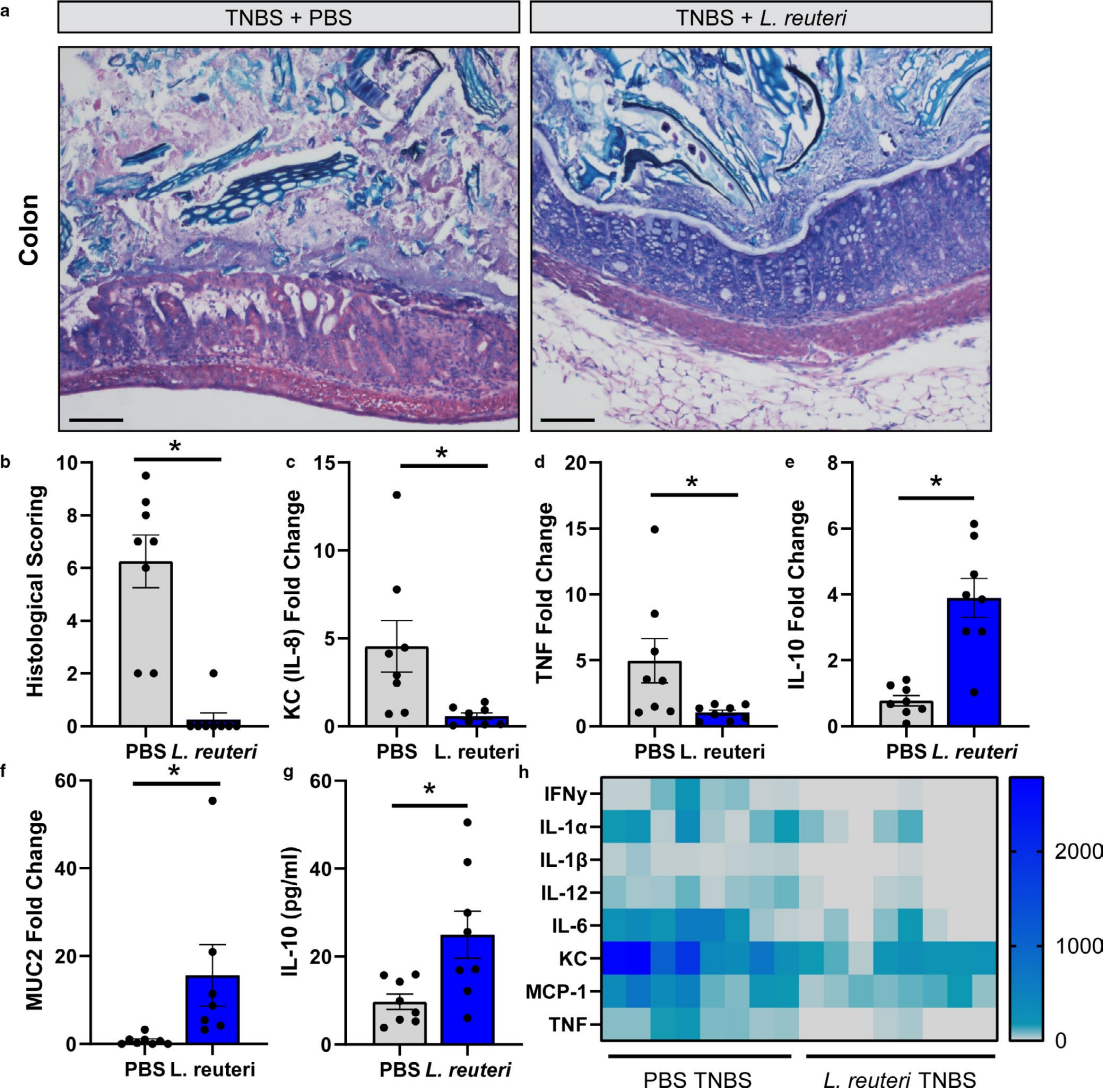
*L*. *reuteri* promotes IL‐10 production and suppresses inflammation in vivo. a. Representative Giemsa stains of mouse colon 3 days following TNBS administration in mice treated with either PBS (control) or 10^9^ live *L*. *reuteri*. Scale bar =100 μm. b. Histological scoring performed by a blinded board‐certified pathologist. qPCR analysis of mRNA of pro‐inflammatory cytokines (c) KC (IL‐8 homolog) and (d) TNF, as well as anti‐inflammatory (e) IL‐10. mRNA analysis of colonic (f) MUC2. Serum anti‐inflammatory IL‐10 (g) and pro‐inflammatory cytokines (h) as assessed by Luminex MAGPIX (*n* = 8 per group; 4 males, 4 females). Student's t‐test, * *p* < 0.05

Our in vitro data support the role of *L*. *reuteri* secreted metabolites and cell components in mediating immune homeostasis. We next sought to define the role *L*. *reuteri* plays in modulating the immune system in the setting of colitis. Intestinal inflammation is accompanied by the infiltration of CD11c^+^ DCs and leukocytes within the lamina propria that secrete greater amounts of pro‐inflammatory cytokines, while producing lower amounts of IL‐10 (Rutella & Locatelli, 2011; Strauch et al., 2010). However, probiotic strains can downregulate pro‐inflammatory mediators and prime Treg cells (Foligne et al., 2007). To address whether *L*. *reuteri* ATCC 6475 could suppress pro‐inflammatory cytokines, BALB/c mice were treated with *L*. *reuteri* or PBS by oral gavage and then TNBS was rectally administered. Immune cells were identified by Giemsa staining (Figure 5A). Increased immune infiltration was observed in PBS treated mice receiving TNBS. Additionally, PBS‐treated mice exhibited loss of goblet cells and the mucus layer as well as alterations in crypt architecture. In contrast, mice treated with 10^9^ CFU *L*. *reuteri* exhibited fewer infiltrating immune cells, retention of goblet cells, and maintenance of crypt architecture. These findings were reflected by the histological scoring (Figure 5B), which indicated that *L*. *reuteri* treated mice overall had less pathophysiology than PBS‐treated mice. The histology was consistent with lower mRNA expression of pro‐inflammatory KC and TNF (Figure 5C,D) and greater mRNA levels of anti‐inflammatory IL‐10 (Figure 5E). Additionally, we observed increased goblet cell MUC2 (Figure 5F). The colonic gene expression was likewise reflected in serum cytokine concentrations, which revealed elevated levels of anti‐inflammatory IL‐10 (Figure 5G) and reduced pro‐inflammatory cytokines in *L*. *reuteri* treated mice compared to PBS treated mice (Figure 5H). These data indicate that *L*. *reuteri* ATCC PTA 6475 is capable of immunomodulation in vivo.

## 4 DISCUSSION

As a major component of the human gut microbiota, *Lactobacilli* generate immunomodulatory effects and can interact with antigen‐presenting cells (Braat, van den Brande, et al., 2004; Braat, de Jong, et al., 2004; Cai et al., 2016; Christensen et al., 2002; Elawadli et al., 2014; Evrard et al., 2011; Fink et al., 2007; Forsythe et al., 2007; Gorska et al., 2014; Haileselassie et al., 2016; Hart et al., 2004; Hsieh et al., 2016; Jo et al., 2016; Jones & Versalovic, 2009; Kagnoff & Eckmann, 1997; Karlsson et al., 2004; Kelsall & Strober, 1997; Ma et al., 2004; Mohamadzadeh et al., 2005; Mowat et al., 2003; Rescigno et al., 2001; Sansonetti, 2004; Tang et al., 2015; Yang et al., 2015; Zeuthen et al., 2006). Select *lactobacilli* stimulate the polarization of naïve immune cells, thereby modifying the immune response (Sudo et al., 1997). The beneficial activity associated with probiotics may be exerted in part through the immunomodulation of gut‐associated lymphoid tissue. Herein we provide evidence that *L*. *reuteri*‐secreted factors stimulate DC maturation, at the level of phenotype and function. While DCs are well versed in responding to pathogens, DC maturation triggers are not unique to infection. DCs are exquisitely sensitive to the intestinal environment and we now recognize that multiple layers of suppression are required to prevent aberrant immune activation (Hammer & Ma, 2013). Modulation of DC responses by commensal bacteria represents a unique avenue to control host inflammation. Several studies have pointed to the anti‐inflammatory properties of commensal gut microbes and probiotics (Guarner & Schaafsma, 1998; Heyman & Menard, 2002; Klaenhammer & Kullen, 1999; Majamaa & Isolauri, 1997). We hypothesize that as a commensal microbe, *L*. *reuteri* adheres to the intestinal mucus layer and secretes metabolites in proximity to the epithelium. We speculate that metabolites transverse the epithelium and activate immature DCs, promoting functional maturation. Induction of IL‐10 producing DCs, in turn, may participate in suppressing inflammation. As overproduction of pro‐inflammatory cytokines has been implicated in chronic inflammatory conditions, suppression of inflammation by *L*. *reuteri* metabolites and surface components may provide a novel therapeutic strategy.

While much is known regarding bacterial‐induced anti‐inflammatory immune responses, few studies have addressed the specific role of bacterial metabolites in immune modulation. Ménard and colleagues previously identified that lactic acid bacterial metabolites could cross the intestinal epithelial barrier and exert anti‐TNF effects on underlying immune cells (Menard et al., 2004). Hoarau *et* *al*. found that the supernatant of *Bifidobacterium* *breve* C50 suppressed LPS‐driven cytokine production in DCs (Hoarau et al., 2006). Previously work from our lab demonstrated that *L*. *rhamnosus* GG conditioned media decreased TNF production by macrophages and *L*. *reuteri* conditioned media decreased TNF production by monocytes; both via a contact independent mechanism (Pena & Versalovic, 2003; Thomas et al., 2012). As the majority of gastrointestinal bacteria reside in the colon, secreted factors that pass through the epithelial barrier to the lamina propria may have a significant impact on local colonic inflammatory responses. Additionally, it has been proposed that the presence of probiotics in the upper gastrointestinal (GI) tract may provide additional anti‐inflammatory signals in an area that typically harbors fewer bacteria (Menard et al., 2004).

Although we are uncertain which metabolites of *L*. *reuteri* activate DCs, there are several potential candidates from the literature. Short‐chain fatty acids (SCFAs) produced by the bacterial fermentation of dietary fibers have been demonstrated by other groups to exert inhibitory effects on cytokine production in immune cells. In macrophages, SCFAs inhibit NFκB activation in the presence of TNF (implications for Crohn's disease, 2000). SCFAs also modulate the production and release of chemokines in neutrophils in the presence of LPS (Vinolo et al., 2009, 2011). Cox *et* *al*. found that SCFAs, of which butyrate was the most and acetate the least potent, inhibit macrophage chemoattractant protein‐1 (MCP‐1, CCL2) in the presence of LPS (Cox et al., 2009). *L*. *reuteri* produces propionate and acetate, and to a lesser degree butyrate (Kahouli et al., 2015). As a result, SCFAs may be responsible for the suppression we observed, but this has yet to be determined. *L*. *reuteri* also produces significant concentrations of lactic acid. Gram‐positive lactic acid bacteria have a cell wall comprised of peptidoglycan and a phospholipid membrane containing lipoproteins, which may be found in small amounts in conditioned media and may activate TLR2. Cell surface proteins from *L*. *reuteri* and *L*. *casei* bind the C‐type lectin DC‐specific intercellular adhesion molecule 3‐grabbing non‐integrin (DC‐SIGN) and promote functional maturation (Smits et al., 2005). *Bifidobacterium* *breve* induces TLR2 and promotes DC maturation. Consistent with this study, we also find that the direct application of UV‐irradiated *L*. *reuteri* bacteria drives DC maturation. It is possible that components of these proteins are found in *L*. *reuteri* conditioned media, which may mediate DC maturation. In the future, we propose to further identify secreted compounds responsible for DC modulation.

In addition to secreted products, *L*. *reuteri* cell surface components were found to stimulate DC maturation. Particularly in terms of IL‐10 production, heat‐killed *L*. *reuteri* were the most potent stimulators. The cell wall of *Lactobacilli* consists of peptidoglycan, decorated with teichoic acids, pilli, exopolysaccharides, and other proteins (Sengupta et al., 2013). These macromolecules are hypothesized to play a major role in determining the species and strain‐specific characteristics of *Lactobacilli*. Lipoteichoic acids (LTA) can bind to TLR2 on antigen‐presenting cells and activate cytokine release (Chapot‐Chartier & Kulakauskas, 2014; Wells, 2011). LTAs from *L*. *fermentum* YIT 0159 and *L*. *casei* YIT 9029 have been shown to activate TLR2 and induce TNF secretion from murine macrophages (Matsuguchi et al., 2003). In another study, purified LTA from *L*. *acidophilus* stimulated IL‐12 and TNF secretion in bone marrow‐derived DCs, while a ltaS mutant deficient in LTAs had reduced abilities to promote cytokine production (Mohamadzadeh et al., 2011). Pilli on *Lactobacilli* are also recognized by DCs. The *L*. *rhamnosus* GG SpaCBA pilli was found to be recognized by C‐type lectin receptor DC‐SIGN and modulated cytokine responses in human DCs (Ossowski et al., 2013; Tytgat et al., 2016). Finally, exopolysaccharides, which can be attached to the cell wall or secreted into the milieu, can be recognized by one of the several C‐type lectin receptors on dendritic cells (Wells, 2011). These components of the *L*. *reuteri* cell wall may be responsible for the effects we observed in our study. Future experiments are warranted to investigate the exact role of these components on *L*. *reuteri*‐induced DC maturation.

One of the characteristic hallmarks of DC maturation is cytokine production. Previous studies have demonstrated that *Lactobacilli* induce distinct strain‐specific DC maturation patterns, with increased levels of cytokines (Christensen et al., 2002; Weiss et al., 2011; Zeuthen et al., 2006). Treatment of immature mouse bone marrow‐derived DCs with lyophilized *L* *actobacilli* were shown to increase the production of TNF, IL‐12, IL‐6, and IL‐10 (Weiss et al., 2011). In this study, *L*. *acidophilus* (strains NCFM, X37), *L*. *casei* (CHCC3137, D12, F19, Nikka, 61R3, 8E2), *L*. *gasseri* (123), *L*. *paracasei* (A14, B32, CRL431, L84, CRL431, Q85, Z11), *L*. *paraplantarum* (D13), *L*. *plantarum* (299v, M.1.1, Q47, 112), *L*. *reuteri* (E14, M.7.1), *L*. *rhamnosus* (19070–2, E5, G26, GG), and *L*. *ruminus* (Q95) were all found to increase DC cytokines, but the cytokine levels varied depending on the strain. In a separate study, the addition of *L*. *rhamnosus* *GG* bacteria to bone marrow‐derived DCs was found to elevate TNF, IL‐12, and IL‐10 (Cai et al., 2016). These cytokine stimulating effects have also been observed in human DCs. *L*. *casei* *Shirota* induced TNF, IL‐12, INFy, and TGF‐β in human blood DCs derived from aged patients (You et al., 2014). *L*. *rhamnosus* Lcr35 was also found to induce TNF, IL‐1β, IL‐12p70, IL‐12p40, and IL‐23 in human blood DCs (Evrard et al., 2011). In these studies, in vitro cytokine production is thought to mirror DC maturation and regulatory cytokine production, rather than a pro‐inflammatory cytokine response.

Our work demonstrates that *L*. *reuteri* elicits the secretion of the anti‐inflammatory cytokine IL‐10 in vivo in TNBS‐treated mice. IL‐10 is proposed to be a master regulator of the immune system (Couper et al., 2008) and during infection, IL‐10 inhibits Th1 cells, NK cells, and macrophage activity. Various cell types can produce IL‐10, including macrophages, DC, B cells, and CD4^+^ or CD8^+^ T cells (Kamanaka et al., 2006). DCs have the unique ability to activate and influence the functional differentiation of naïve T cells and drive cells toward IL‐10 production. In animal and human models, select probiotic bacteria enhance IL‐10 production from PBMCs, splenocytes, Peyer's cells, and DCs (Lammers et al., 2003; Roller et al., 2004; Schultz et al., 2003). Additionally, in human DCs, *L*. *reuteri* and *L*. *casei*, but not *L*. *plantarum*, primed DCs to drive the development of Treg cells (Smits et al., 2005). These Treg cells produced increased concentrations of IL‐10 and inhibited the proliferation of bystander T cells in an IL‐10‐dependent manner (Smits et al., 2005). We speculate that *L*. *reuteri* may prime DCs in vivo to drive Treg IL‐10 production. However, *L*. *reuteri* may also directly modulate T cell activity, which has been previously demonstrated (Braat, van den Brande, et al., 2004; Chapat et al., 2004; Kruisselbrink et al., 2001; Liu et al., 2013; Mohamadzadeh et al., 2005; Peluso et al., 2007; Pessi et al., 2001).

## 5 CONCLUSIONS

Our work indicates that *L*. *reuteri* surface components and secreted metabolites promote DC maturation and production of anti‐inflammatory IL‐10. Moreover, *L*. *reuteri* administrator diminishes inflammation a model of acute colitis. Collectively, the data presented herein provide further support for the beneficial role of *L*. *reuteri* secreted products in immune modulation in vitro and in vivo and in the suppression of inflammation.

## DISCLOSURES

JV receives unrestricted research support from the Swedish Probiotic Company BioGaia AB. JV serves on the scientific advisory boards of Seed, a USA‐ based probiotics/prebiotics company, Biomica, an Israeli informatics enterprise and Plexus Worldwide, a USA‐based nutrition company. All other authors have no relationships to disclose.

## AUTHOR CONTRIBUTIONS

Concept and design (MAE, JV); intellectual contribution (MAE, WR, RF, ZS, CV, JV); data acquisition (MAE, WR, ME, RF, ZS, KAE, ACE, FDI, CV, SV, DAS); data analysis, statistics, and interpretation (MAE, WR, RF, ZS, KAE, ACE, FDI, CV, SV, DAS); drafting and editing manuscript (MAE, WR, RF, ZS, KAE, ACE, FDI, CV, SV, DAS, JV); funding (JV).
